# Towards goal-directed perfusion - Part II: Temperature dependence of Q_10_, oxygen-demand dependence on age and weight, further clinical insights

**DOI:** 10.1177/02676591251385838

**Published:** 2025-10-14

**Authors:** Mansour T. A. Sharabiani, Alireza S. Mahani, Richard W. Issitt, Yadav Srinivasan, Alex Bottle, Serban Stoica

**Affiliations:** 1 School of Public Health, 4615 Imperial College, London, UK; 2 New York Stock Exchange, New York, USA; 3 Centre for Heart Failure, Transplantation and Extracorporeal Support, UCL Institute for Cardiovascular Science, 4919 UCL, London, UK; 4 Perfusion Department, 4956 Great Ormond Street Hospital for Children, London, UK; 5 Data Research, Innovation and Virtual Environments Unit, 4956 Great Ormond Street Hospital for Children, London, UK; 6 Cardiac Surgery Department, 4956 Great Ormond Street Hospital for Children, London, UK; 7 Cardiac Surgery Department, 156596 Bristol Royal Children’s Hospital, Bristol, UK

**Keywords:** cardiopulmonary bypass, van’t Hoff, oxygen delivery, generalised additive models, system dynamics

## Abstract

**Background:**

Goal-directed perfusion (GDP) during cardiopulmonary bypass (CPB) seeks to match oxygen delivery with patient-specific metabolic demand, yet determinants of oxygen demand and dynamics of oxygen extraction ratio (OER) are not well characterised in paediatric populations. Building on our GARIX (Global AutoRegressive Integrated model with eXogenous variables and an equilibrium force) model from Part 1, we analysed minute-level intraoperative data to derive clinical insights.

**Methods:**

GARIX integrates autoregressive memory, exogenous clinical variables (cardiac index, haemoglobin, arterial oxygen saturation, temperature), and an equilibrium component defining indexed oxygen demand (tVO_2_i) as a function of age, weight, and temperature. We applied GARIX to 20,443 minutes of data from 293 paediatric CPB procedures to study: (1) age- and weight-related variation in oxygen demand; (2) temperature dependence of demand, estimating local and interval Q_10_; (3) age-stratified OER responses to haemoglobin and other variables; and (4) time evolution of the mismatch between oxygen consumption and demand after step changes in oxygen delivery.

**Results:**

tVO_2_i followed a non-monotonic pattern, rising from birth to ∼3 years and declining thereafter. Metabolic demand decreased nonlinearly with temperature, with a 17% reduction from 37°C to 32°C and 39% further reduction to 27°C. Local Q_10_ ranged from ∼1.3 near normothermia to ∼3.8 in deep hypothermia. OER responsiveness to haemoglobin was blunted in neonates and infants compared with older children. Simulations revealed transient over- and under-oxygenation following step changes in oxygen delivery due to lags in OER adaptation.

**Conclusions:**

We identify risks of over- and under-oxygenation during CPB despite normal OER readings, challenge the use of fixed targets and constant Q_10_, and highlight age- and weight-dependent dynamics. These findings support individualised, time-aware perfusion strategies and lay the groundwork for model-guided decision support and control.

## Introduction

The Global AutoRegressive Integrated time-series model with eXogenous variables and an equilibrium force (GARIX), introduced in Part 1 of this series, provides a physiology-inspired framework for modelling oxygenation dynamics during paediatric cardiopulmonary bypass (CPB). GARIX predicts minute-by-minute changes in the oxygen extraction ratio (OER) by integrating three forces: (1) autoregressive memory of past OER values, (2) the effect of recent - and intended - changes in exogenous – or controllable – inputs, namely cardiac index (CI), haemoglobin concentration in the blood (Hb), oxygen saturation of arterial haemoglobin (SaO_2_) and body temperature (Temp), and (3) a corrective equilibrium force aimed at aligning oxygen consumption with temperature- and patient-specific metabolic demand. The oxygen demand model of GARIX extends the classical van’t Hoff specification^1^ by using a nonlinear spline function for temperature effects and by allowing baseline metabolic demand to vary with age, weight, and their interaction. These features enable GARIX to accommodate inter-patient heterogeneity and nonlinear metabolic suppression during hypothermia.

Understanding the dynamics of OER and oxygen demand is clinically vital for implementing goal-directed perfusion (GDP). OER mediates the relationship between oxygen delivery and consumption and serves as a critical indicator of perfusion adequacy. Yet, during CPB, OER is not directly controllable and may react with significant delay to sudden changes in flow or oxygen content. This creates the potential for transient over- or under-oxygenation. Moreover, it is reasonable to suspect that oxygen demand is not uniform across patients - it varies with developmental stage, body size, and temperature - and perfusion targets that ignore this variability risk oversupplying or undersupplying key organs. Quantitative models like GARIX can help identify, interpret, and ultimately prevent such mismatches.

In this second part, we apply GARIX to high-resolution intraoperative data to generate clinical insights. We begin by comparing GARIX-predicted metabolic rates to established equations, and by examining how oxygen demand varies with age and weight. We then analyse age-related differences in OER responsiveness to changes in haemoglobin and other exogenous variables. Next, we quantify the nonlinear dependence of oxygen demand on temperature and derive local and interval Q_10_ values. Finally, we use GARIX-based simulations to study oxygenation gap (OG) dynamics following step changes in oxygen delivery. Collectively, these analyses highlight the limitations of uniform and static perfusion strategies and underscore the need for physiology-aware, individualised targets during CPB.

For a list of nonstandard abbreviations and acronyms used in this manuscript, see Supplemental Material A.

## Methods

The statistical modelling framework used in this manuscript is GARIX. Its analytical foundations including the definitions of various term groups (TGs), concept of equilibrium analysis, modelling of oxygen demand, and system simulations using GARIX, are described in Part 1 of this series, and further explained in Supplemental Material D.

### Study design and patient cohort

This study uses the same paediatric CPB dataset described in Part 1, consisting of minute-by-minute intraoperative data collected from 963 procedures performed during 2019 to 2021 at the Great Ormond Street Hospital. As in Part 1, we restricted model training to a subset of 293 procedures from 286 patients who did not develop postoperative acute kidney injury (AKI), to focus on physiological patterns and oxygenation targets under presumed normal adaptive function.

**Figure 1.**
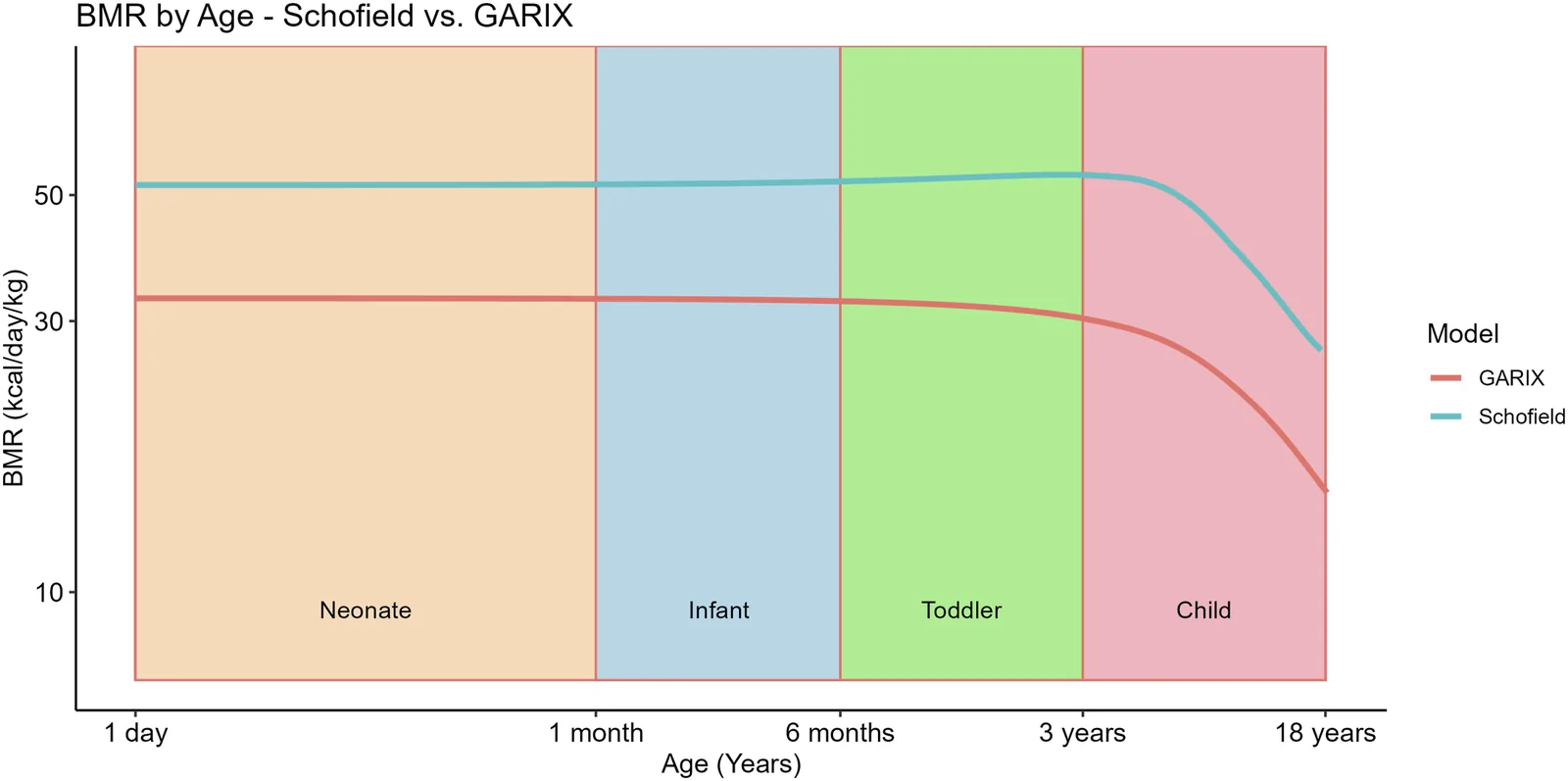
BMR predictions – per kilogram of body weight – according to GARIX and Schofield as a function of age, at Temp = 37°C. Each data point corresponds to a patient’s age and weight in our dataset (including those with AKI). LOESS fits to each dataset are overlaid. Note that *x* and *y* axes are displayed on a logarithmic scale.

**Figure 2.**
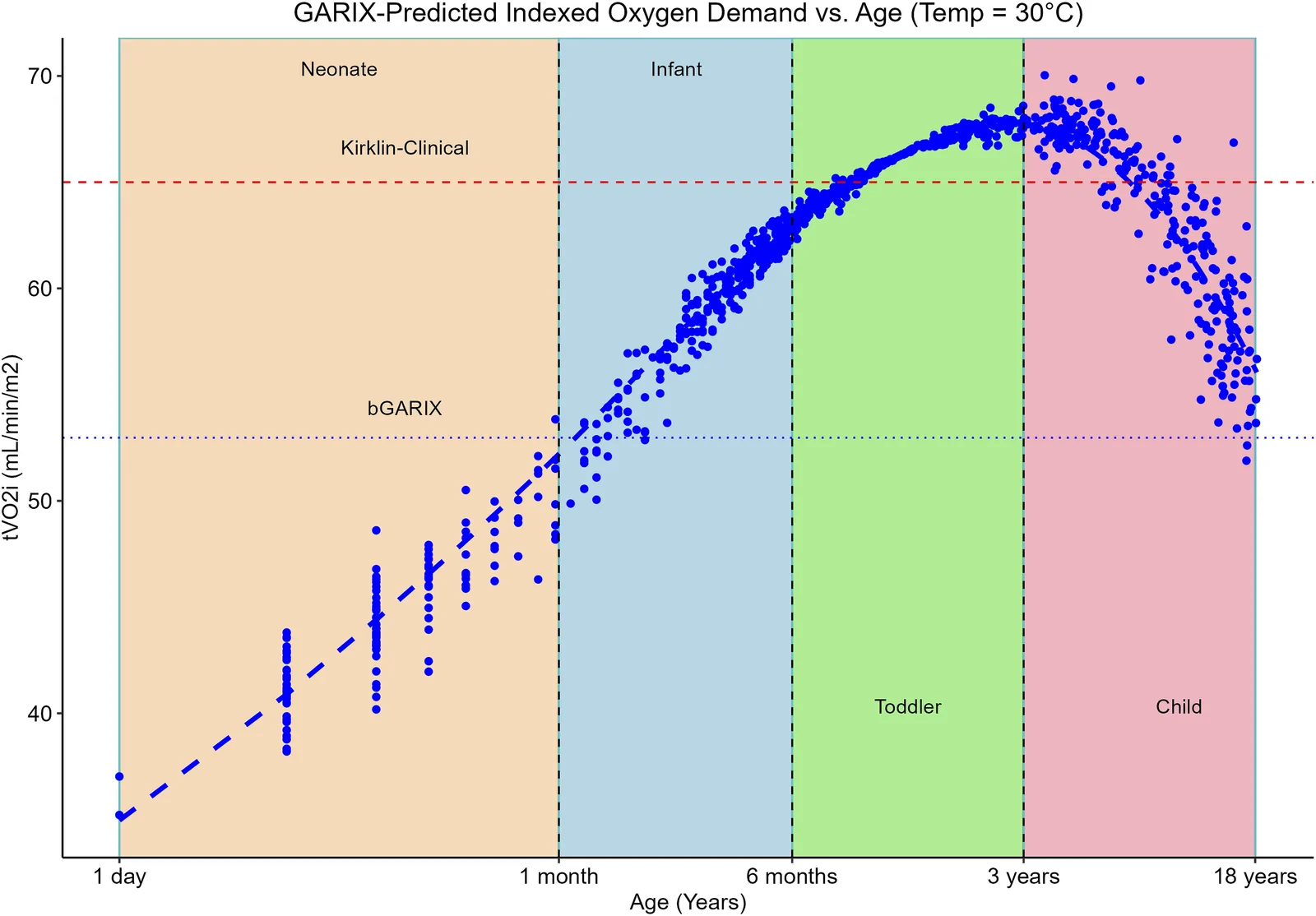
GARIX-predicted tVO_2_i at Temp of 30°C versus patient age. Model predictions were calculated for all (age, weight) pairs in our data and projected onto the age-tVO_2_i plane (red dots). Green line is a nonparametric (loess) regression line. Colour-shared areas represent neonate (orange), infant (blue), toddler (green) and child (pink) age groups. Dotted blue line is the bGARIX prediction, which is independent of age and weight. Dashed red line (‘Kirklin-Clinical’) is taken from Figure 2.11 of^1^ (small X’s on the hyperbolic curves).

However, two analyses in this manuscript leveraged the full dataset of 963 patients. First, we compared predicted metabolic rates from GARIX with those from the Schofield equations, using all available (age, weight) pairs to enable robust benchmarking across developmental stages (Figure 1). Second, we visualised GARIX-predicted indexed oxygen demand (tVO_2_i) as a function of age by evaluating the model at each observed (age, weight) combination, producing a population-wide age profile of metabolic demand (Figure 2). In both cases, inclusion of the full cohort allowed for more reliable characterisation of physiological variation during childhood.

Age was treated both continuously and categorically, with the latter consisting of four age groups: neonates (<1 month), infants (1–6 months), toddlers (6 months–3 years), and children (>3 years).

### Modelling oxygen demand by age and weight

To evaluate the influence of age and weight on oxygen demand, we used equilibrium analysis of GARIX (Supplemental Material D) to infer tVO_2_i across the population. Under equilibrium conditions – where OER and its predictors remain stable – the model implies that indexed oxygen consumption (VO_2_i) equals the internally modelled target (tVO_2_i), which can therefore be interpreted as latent (indexed) metabolic demand (indexing is done using patient’s body surface area or BSA. See Supplemental Material B). GARIX includes terms for age, weight, and their interaction, enabling flexible, non-monotonic relationships. For empirical visualisation, oxygen demand was predicted at each patient’s observed age and weight, predictions were projected onto the age axis, and locally estimated scatterplot smoothing (LOESS) was applied to the resulting projections.

To benchmark against established clinical standards, we first converted GARIX-predicted indexed oxygen consumption to (unindexed) oxygen consumption, by multiplying the former by the BSA of each patient. We then converted oxygen consumption (mL O_2_ min^−1^) to energy expenditure (kcal day^−1^) using the common caloric equivalent of ∼5 kcal per litre of O_2_.^2^ Because 1 mL O_2_ min^−1^ equals 1.44 L O_2_ day^−1^, the appropriate conversion factor is 1.44 L × 5 kcal L^−1^ ≈ 7.2 kcal day^−1^ per (mL O_2_ min^−1^).

### Subgroup analysis of OER dynamics

To investigate age-related differences in oxygen extraction dynamics, we trained separate GARIX models within each age group and extracted the lagged-difference coefficients for Hb and other exogenous variables (CI, SaO_2_ and Temp). These coefficients quantify the marginal effect of changes in each variable (i.e., while keeping other variables unchanged) at different time lags on the logit of predicted OER, where logit (OER) = log (OER/(1 - OER)).

### Modelling temperature dependence of oxygen demand

The equilibrium term group (ETG) in GARIX includes a nonlinear temperature response using spline functions and incorporates patient-specific factors (logarithm of age and weight, and their interaction). The resulting oxygen demand model (Supplemental Material D) was used to predict tVO_2_i over a finely spaced grid of 100 temperature values spanning the observed range and for all combinations of age and weight in our data.

Uncertainty was quantified with 1000 non-parametric bootstrap replications in which complete patient procedures were resampled, a new GARIX model refitted, and tVO_2_i re-predicted over the grid. 95% confidence intervals (CIs) were taken from the 2.5 % and 97.5 % bootstrap quantiles. Temperature-specific metabolic sensitivity was summarised with (i) local Q_10_ values – reflecting the instantaneous relative change in demand per 10°C – and (ii) interval Q_10_ values anchoring normothermia at 37°C. Both metrics were calculated for GARIX, as well as a global Q_10_ using the baseline GARIX (bGARIX) model. For details on how local and interval Q_10_ was calculated for GARIX, see Supplemental Material D.

Past Q10 values reported in literature – using human and animal experiments – were added for comparison. For details on the textbook models of Kirklin as well as its clinical recommendations, see Supplemental Material G.

### OG simulation

To assess how mismatches between oxygen supply and demand evolve over time, we conducted deterministic simulations using GARIX under step changes in indexed oxygen delivery (DO_2_i), induced by controlled modifications to CI. OG was defined as the percentage deviation of actual oxygen consumption (VO_2_i) from its target (tVO_2_i): OG = 100 × (VO_2_I - tVO_2_i)/tVO_2_i. Supplemental Material D describes GARIX simulations in full detail.

## Results

### Oxygen demand by age

Figure 1 compares age-wise predictions of basal metabolic rate (BMR) per kilogram of body weight, using GARIX and Schofield equations.^3^ Looking at the two curves, one can see they follow similar patterns, which suggests that despite differing estimation methods and data sources, GARIX captures age-dependent metabolic patterns in a manner broadly consistent with Schofield equations. This is an independent empirical support for GARIX. The difference in the intercept – despite the overall similarity of the patterns – may be the reflection of context-specific differences during CPB, such as the effects of muscle relaxation.^4^

Figure 2 projects GARIX-predicted tVO_2_i – at 30°C – onto the age axis, evaluated at all (age, weight) pairs in the training data. A clear non-linear trend emerges: tVO_2_i increases in neonates and infants, peaks around 3 years, and subsequently declines in older children. A uniform tVO_2_i across childhood – implied by the bGARIX and ‘Kirklin-Clinical’ lines in Figure 2 – may lead to significant under- and over-estimation of oxygen demand across paediatric age groups. For instance, while Kirklin’s clinical recommendation is close to GARIX for small children, it would lead to significant over-oxygenation for neonates and infants.

We also note that the coefficient of the interaction term between log (age) and log (weight) in the ETG of GARIX is statistically significant, which implies that the dependence of oxygen demand on patient weight varies by age. See Supplemental Material F.

### Age-dependent response to Hb Change

**Figure 3.**
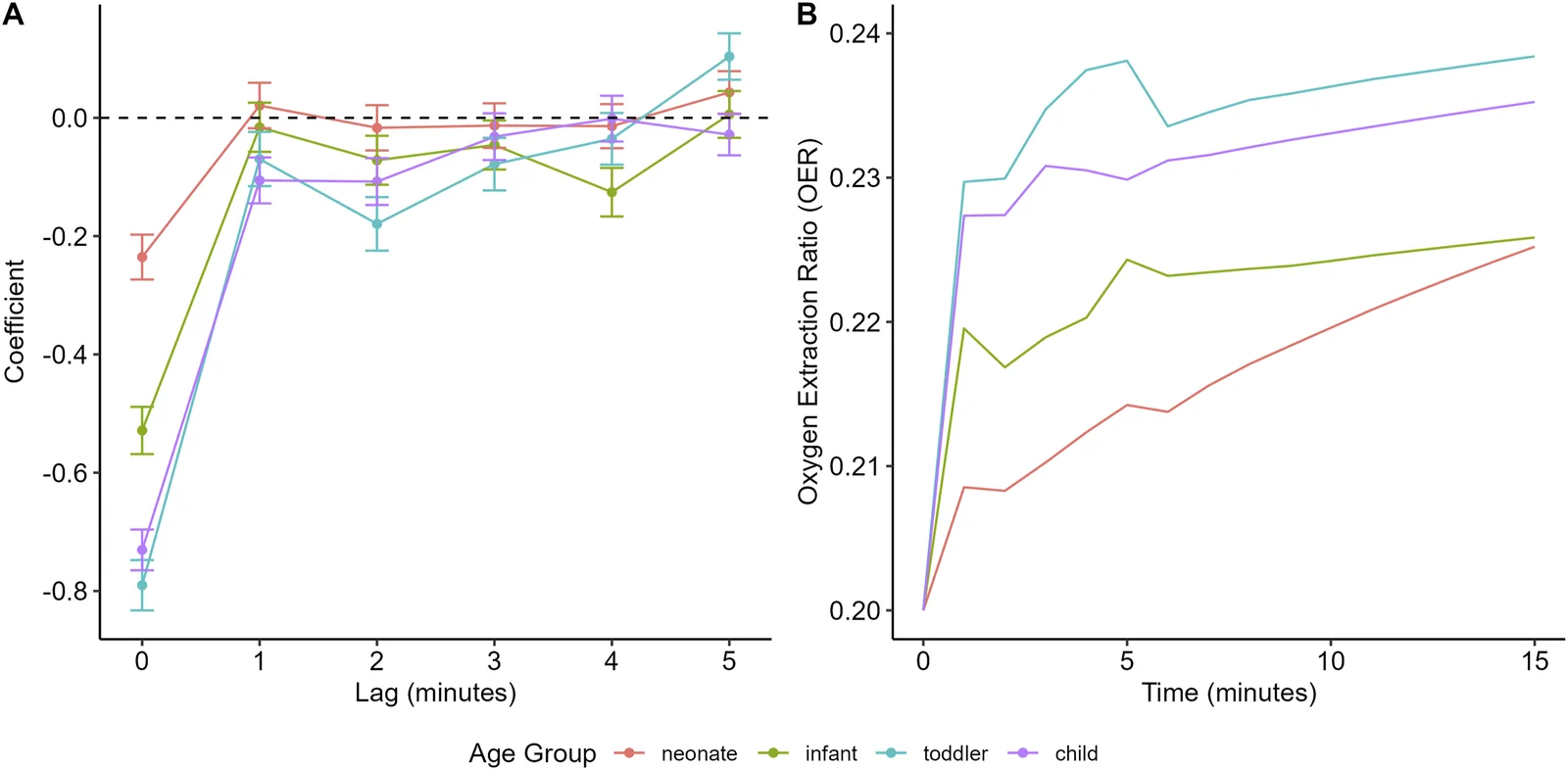
OER Reaction to Hb Change by Age Group. A: Coefficients of change terms in GARIX (5) involving haemoglobin (log (Hb)), fitted to subsets of training data in each age group. B: OER trajectory within 15 minutes of a step reduction in Hb according to each age-group-specific model. Within each age group, age and weight are chosen to be medians of data. CI, SaO_2_ and Temp are global medians of the data. Initial and final Hb in each age group is chosen to obtain initial and final OER values of 0.20 and 0.25, respectively.

Figure 3(a) shows the GARIX-estimated coefficients for the lagged-difference terms of log (Hb), stratified by age group. The impact of haemoglobin changes on OER is strongest at lag 0 and diminishes with increasing lag, consistent with a short-term physiological adaptation (as discussed in Part 1). However, the magnitude of this immediate response varies by age, with older children exhibiting a coefficient of larger magnitude than neonates and infants. This difference in the strength of coefficients manifests itself in slower rate of immediate OER reaction to Hb change across age groups (Figure 3(b)), with younger patients showing a much slower OER response within the first few minutes of Hb change.

Supplemental Material F shows similar plots of age-stratified, lagged-difference coefficients for CI, Temp, SaO_2_ and OER.

### Temperature-dependence of oxygen demand

**Figure 4.**
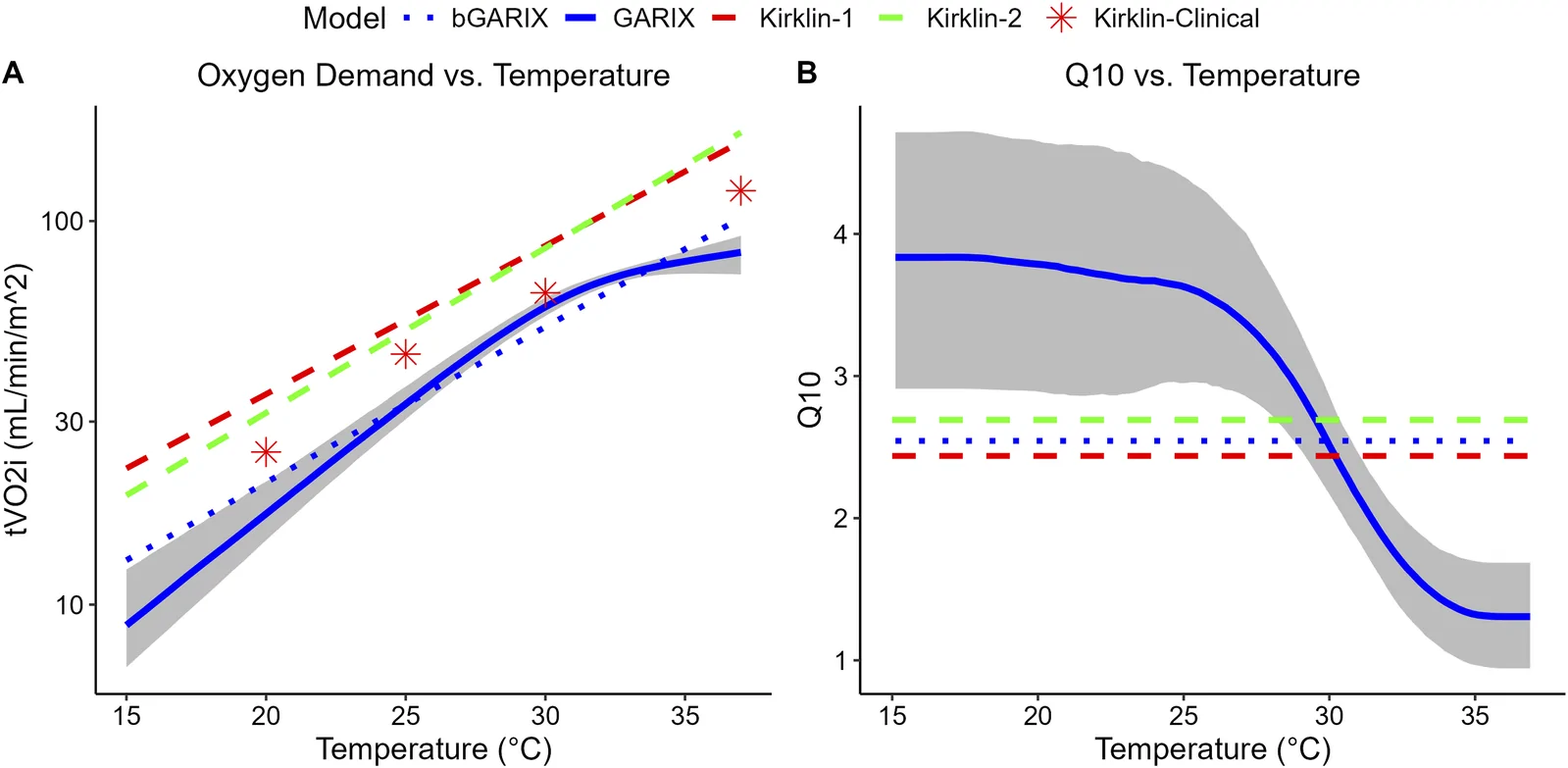
Oxygen demand versus Temperature. A: Estimated tVO_2_i versus temperature, with each point being the geometric mean of GARIX-predicted values for all (age, weight) combinations in training data. Dotted blue lines represent predictions from bGARIX which form a straight line given the logarithmic scale of the *y* axis (van’t Hoff). ‘Kirklin-1’ (red dashed line) is taken from^1^ (Equation 2A-3) by setting cardiac index to infinity (asymptotic consumption). ‘Kirklin-2’ is also taken from^1^ (Equation 2A-1), after converting tVO2i from per kilogram of weight to per m2 of BSA using an average ratio extracted from our data. ‘Kirklin-Clinical’ is taken from Figure 2.11 of^1^ (small X’s on the hyperbolic curves). B: Same sources as A, but *y* axis represents Q_10_. In the case of GARIX, it represents ‘local’ Q_10_.

Figure 4(a) displays the GARIX-predicted tVO_2_i (on a logarithmic scale) as a function of temperature. A clear nonlinearity is observed: oxygen demand decreases modestly with mild hypothermia but below 30°C it drops sharply. For example, reducing temperature from 37°C to 32°C yields only a 17% reduction in demand, whereas the next 5°C decrease leads to a 39% reduction. The overall levels of predicted oxygen demand by GARIX are below the two models listed in the Kirklin textbook^1^ (see Supplemental Material G). It is noteworthy, however, that the ‘clinical’ oxygen demand targets recommended in Kirklin are closer to GARIX predictions, and the two are indeed very close in the mild hypothermic region, where most of our intraoperative recordings – and likely in the broader CPB operations – are gathered (see Figure 3(b) in Part 1).

Figure 4(b) illustrates the temperature dependence of Q_10_ – according to GARIX – more vividly: Q_10_ falls from 3.8 at 16°C to approximately 1.3 near 37°C, indicating that the metabolic rate becomes less temperature-sensitive near normothermia. Nevertheless, the global Q_10_ value predicted by bGARIX is 2.5, aligning closely with the textbook values of 2.4 and 2.7 mentioned in Kirklin.^1^

**Figure 5.**
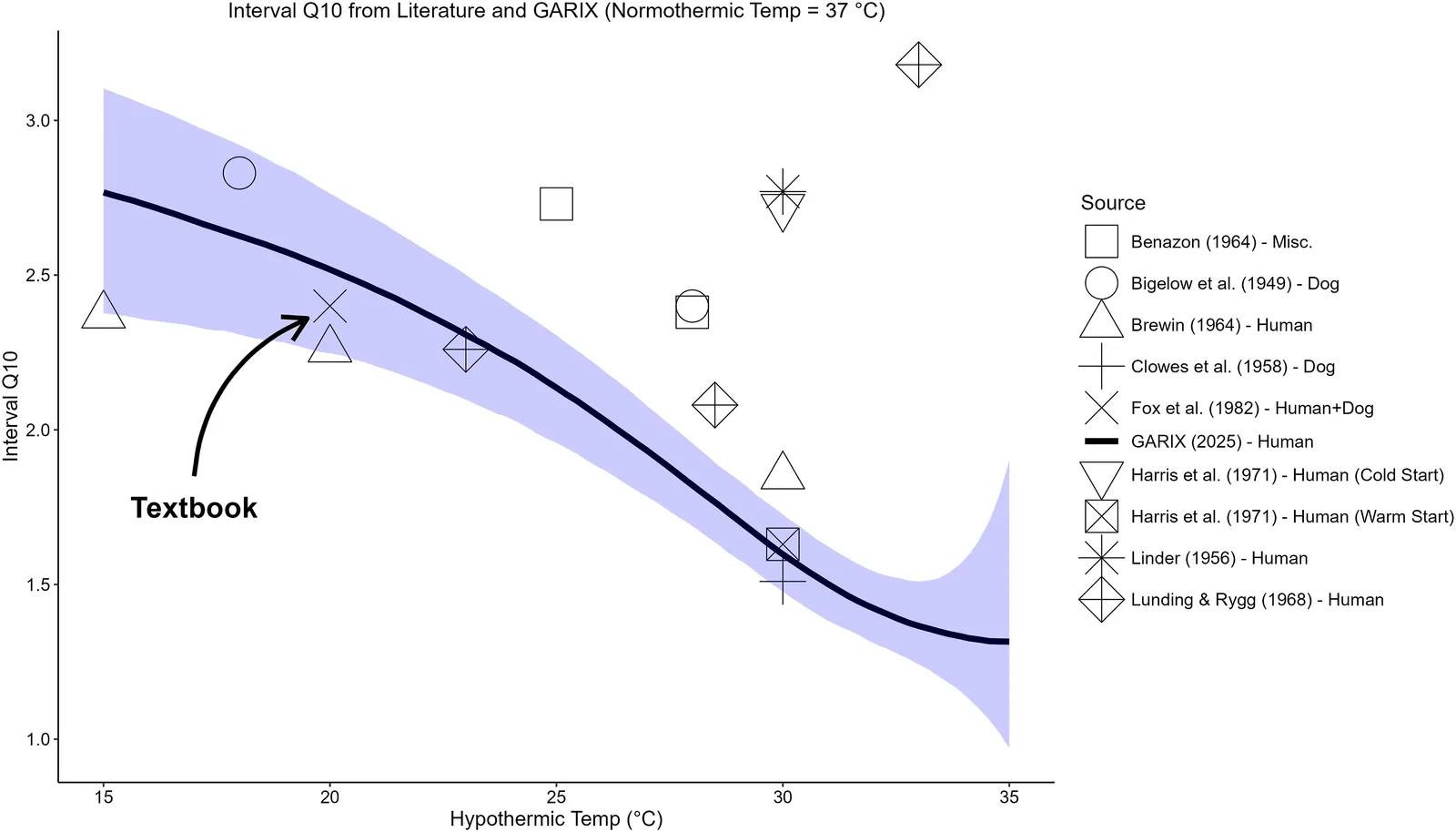
Comparison of interval Q_10_ estimated by GARIX against literature. The normothermic end of the interval is 37°C, and the *x*-axis shows the hypothermic end of the interval. References are Benazon (1964),^5^ Bigelow et al. (1949),^6^ Brewin (1964),^7^ Clowes et al. (1958),^8^ Fox et al. (1982)^9^ (listed in Kirklin’s textbook^1^), Harris et al. (1971),^4^ Linder (1956),^10^ Lunding & Rygg (1968).^11^

Figure 5 presents the ‘interval’ Q_10_ values estimated by GARIX, defined relative to a fixed normothermic temperature of 37°C, alongside values reported in the literature. The GARIX-based interval Q_10_ declines with increasing hypothermic endpoint, ranging from approximately 2.7 at 16°C to 1.3 near 37°C. Several historical studies, including those by Brewin (1964), Bigelow et al. (1949) and Benazon (1964), report multiple interval Q_10_ values at different hypothermic endpoints and show similar trends, with higher Q_10_ observed for deeper hypothermia. An exception is Lunding & Rygg (1968) where the value at 33°C appears to be an outlier.

Table 1 summarises the oxygen demand predictions by Kirklin (clinical) and GARIX. Note that GARIX predictions are differentiated by age, while Kirklin figures are not. The ‘% of Normal’ columns show, according to each method, oxygen demand as percentage of its value at normothermia (37°C). While at 20°C the two methods converge (21% for both Kirklin and GARIX), the trajectory at intermediate temperatures differs significantly. For instance, at 30°C Kirklin indicates that demand has nearly halved (54%), while GARIX shows the demand being 72% of normal temperature. These results reflect the non-constant Q_10_ produced by GARIX (smaller values at higher temperatures) versus a (near) constant Q_10_ implied by the textbook clinical recommendations (Figure 4).

**Table 1.** Indexed Oxygen Demand – GARIX versus Kirklin.

Temp (°C)	Kirklin – Clinical		GARIX				
	VO2i (mL/min/m2)	% Of normal	VO2i (mL/min/m2)				% Of normal
			5 days, 3.0 kg	6 months, 6.4 kg	3 years, 13.1 kg	16 years, 54.4 kg	
37	120	**100**	59	88	94	80	**100**
30	65	**54**	42	63	68	57	**72**
25	45	**38**	23	35	38	32	**40**
20	25	**21**	12	18	20	17	**21**
15	-	**-**	6	9	10	8	**11**

Comparison of indexed oxygen demand predictions by GARIX (for representative age/weight values as well as population average) and clinical recommendations in Kirklin^1^ (Figure 2–11). ‘% of Normal’ columns show the predicted oxygen demand by each method as a percentage of the value at normothermia (37 °C). Note that the ‘% of Normal’ column for GARIX applies to any age/weight combination.

## OG dynamics

**Figure 6.**
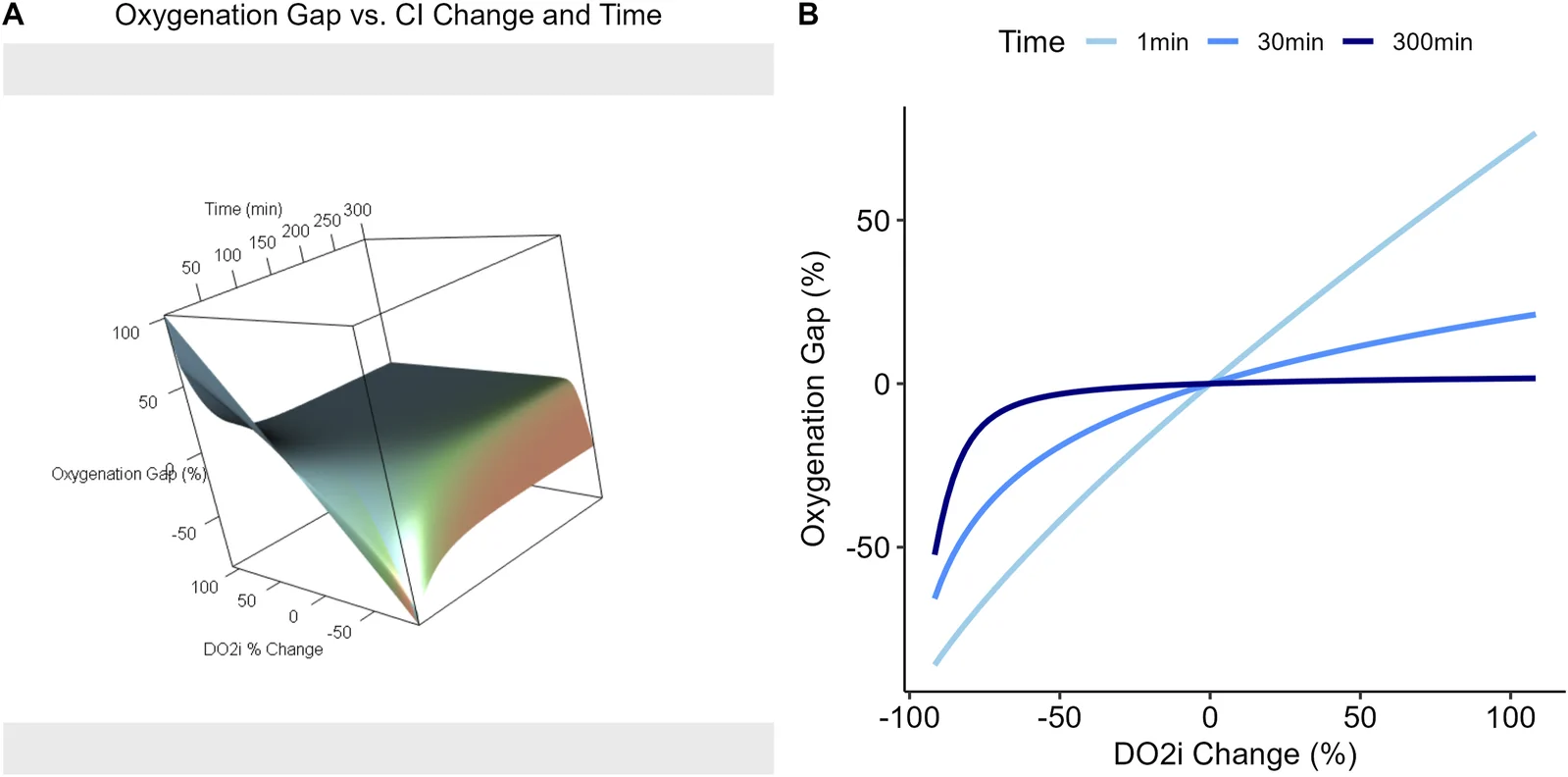
OG as a function of % change in DO_2_i (via a step change in CI at t = 0), at different wait times, using deterministic simulation with a GARIX model. State variables are log (CI) = 0.18, log (Hb) = 1.96, log (SaO_2_) = 4.6, temp = 20, logit (OER) = −1.61. Positive OGs indicate over-oxygenation and negative OGs reflect under-.oxygenation.

Figure 6 illustrates the short-term and long-term behaviour of the OG following a step change in DO_2_i, induced via a controlled change in cardiac index (CI). Immediately after the increase in DO_2_i, the predicted OG becomes transiently positive, indicating over-oxygenation due to a lag in the adaptive response of the OER. Over time, as OER decreases, the gap narrows and approaches zero, representing re-equilibration between oxygen consumption and demand. This dynamic pattern reveals that the body does not instantaneously extract a smaller fraction of oxygen when its supply is elevated, contrary to the assumption of immediate supply-demand matching in static models.

Notably, similar behaviour is observed in the under-oxygenation regime: a decrease in DO_2_i initially causes OG to drop below zero, even if OER is not near critical thresholds and could safely rise to maintain the consumption level.

## Comment

### Key insights

Below is a summary of our key insights in Part 2.

• **Age & Weight Dependence:** Indexed oxygen demand shows a non-monotonic relationship with age, peaking around 3 years, with weight effects varying by age.
• **Temperature Dependence:** Metabolic suppression is nonlinear – modest at mild hypothermia (≈17% drop from 37°C to 32°C) but pronounced at deeper hypothermia (≈39% additional drop), with local Q_10_ values ranging from ∼3.8 (16°C) to ∼1.3 (37°C).
• **OER Response to Hb:** Younger patients exhibit attenuated OER responses to Hb changes, possibly due to stored red blood cell (RBC) effects on oxygen unloading.
• **Dynamic Adaptation:** Step-change simulations show transient over- and under-oxygenation due to delayed OER adaptation, even when OER path is well below critical levels.

### Metabolic suppression during CPB

Variations in metabolic demand by age and weight have long been established, as evidenced by the Schofield equations for predicting BMR.^3^ Indeed, one could have used these equations to derive a tVO_2_i profile across age and weight, similar to our approach for comparing GARIX and Schofield predictions (Figure 1). The observed multiplicative gap between GARIX predictions and Schofield estimates, averaging approximately 58%, is directionally aligned with Harris et al. (1971),^4^ who demonstrated a metabolic suppression following anaesthesia, yielding a ratio of 84% in adult patients. They attributed this suppression primarily to muscle relaxation induced by anaesthesia. The larger implied gap in our paediatric patient population might reflect more pronounced metabolic suppression compared to adults, possibly due to the substantial reduction in growth-related metabolic processes during anaesthesia and CPB. Future studies comparing similar modelling techniques across paediatric and adult populations would be valuable in clarifying the contributions of age-related physiology and CPB management strategies to these observed differences.

### Personalising perfusion by age and body size

Indexed oxygen demand shows a clear non-monotonic relationship with age, rising in early life, peaking around 3 years, and then declining in older children. Weight also modulates demand differently across age groups: in neonates and infants, higher weight is associated with higher oxygen demand, but this effect weakens and eventually reverses in older children. These patterns likely reflect developmental changes in metabolic rate, body composition, and organ maturity, underscoring the need for age- and weight-individualised perfusion strategies.

The approximately 40 mL/min/m^2^ spread – at 37°C – in GARIX-predicted tVO_2_i across childhood represents a substantial physiological gradient - comparable to the difference in oxygen consumption between deep hypothermia and normothermia - and challenges one-size-fits-all perfusion targets. Neonates and older children may require lower indexed flows to match their lower metabolic demands, while toddlers at peak demand may benefit from higher flow-index targets to avoid under-oxygenation.

GARIX-predicted demand surfaces provide a scalable way to personalise these targets. By outputting tVO_2_i for any combination of age, weight, and temperature, GARIX can be embedded into perfusion dashboards or pump consoles, enabling real-time comparison of VO_2_i and tVO_2_i. Such integration could shift practice from protocol-driven to physiology-driven perfusion management, tailored to each patient’s metabolic profile.

Finally, age-related differences in oxygen extraction dynamics further emphasise personalisation: in paediatric CPB, haemoglobin increases often involve transfusion of stored RBCs, more common in younger patients due to circuit volume relative to body size. Stored RBCs may unload oxygen more slowly, reducing O_2_ extraction,^12^ and storage-induced changes can alter the oxygen dissociation curve, affecting SaO_2_–PaO_2_ relationships.^13,14^ Such age-dependent reactions to clinical inputs must be taken into account for optimal perfusion management.

### Re-evaluating temperature management

The GARIX model indicates that relying on constant-Q_10_ tables to guide temperature management during CPB can be misleading, especially in the transition from moderate to deep hypothermia. Constant-Q_10_ assumptions imply a fixed multiplicative change in metabolic rate for every 10°C shift in body temperature, but GARIX reveals a sharp change in Q_10_ around 30°C, where metabolic suppression accelerates nonlinearly and Q_10_ values rise steeply as temperature falls. For example, cooling from 37°C to 32°C produces only modest metabolic suppression (∼17%), whereas an additional drop to 27°C yields a much larger reduction in oxygen demand (∼39%). Overall, Q_10_ varies from ∼1.3 near normothermia to ∼3.8 at 16°C, highlighting a highly temperature-sensitive regime at lower temperatures. These findings suggest that moderate cooling offers limited metabolic benefit, while deeper hypothermia provides disproportionate reductions in oxygen demand – though with potential clinical trade-offs.

GARIX-predicted demand curves also show that, while the average Q_10_ from bGARIX aligns closely with the value reported in Kirklin 1, the absolute levels of oxygen demand are lower. Notably, the clinical values recommended in Kirklin nearly match GARIX predictions in the 30°C range, where most intraoperative data are concentrated. Incorporating empirically derived, temperature-sensitive demand curves such as those from GARIX may therefore enable more precise and adaptive temperature management during CPB. Mechanistically, the observed non-constancy of Q_10_ may reflect the behaviour of allosteric enzymes regulating glycolysis, which often show sigmoid rather than simple van’t Hoff responses to temperature.^15^ Mild hypothermia may maintain sufficient enzymatic function and substrate availability to decouple metabolic rate from temperature, whereas deeper cooling reduces this independence, leading to higher Q_10_ values. Further studies are warranted to validate these findings and clarify the underlying mechanisms.

### Dynamics of OER adaptation and OG

The GARIX-based simulations demonstrate that the OER does not adjust instantaneously to changes in DO_2_i, resulting in transient OGs even when OER path remains below critical levels. The lag in OER adjustment carries important clinical implications. Following a sudden increase in pump flow, for instance, oxygen delivery can temporarily overshoot metabolic demand, leading to brief episodes of cellular hyperoxia. Conversely, a rapid flow reduction may induce a short-lived undersupply, risking cellular hypoxia even if mean OER remains below traditional critical thresholds. In vulnerable patients – such as those with marginal oxygen reserves or impaired autoregulation – even short bouts of under- or over-oxygenation may contribute to tissue injury or reperfusion-related stress.

To mitigate these effects, clinical strategies could aim to dampen the rate of change in oxygen delivery and anticipate the time-dependent nature of OER adjustment. Graduated flow ramps, in place of abrupt step changes, can allow more synchronous adaptation between supply and extraction. Similarly, anticipatory modulation of haemoglobin (e.g., timing transfusions before expected rises in flow) can help buffer transitions, aligning oxygen content more closely with predicted demand during dynamic shifts. Embedding GARIX or similar dynamic models into perfusion dashboards could further support this approach by projecting short-term OGs and guiding real-time intervention to pre-empt imbalance.

### Limitations and future work

The oxygen demand curves generated by GARIX are based on retrospective intraoperative data and reflect average conditions observed in a single-centre setting. While the model accounts for patient-specific variation in age and weight, other important factors – such as congenital heart anatomy, myocardial function, and intraoperative events – are not yet explicitly included. Future work should examine whether incorporating procedural or diagnostic subgrouping can further personalise the demand estimates.

Although GARIX relaxes the constant-Q_10_ assumption via spline-based temperature modelling, the precision of Q_10_ estimates in the deep hypothermia range remains limited by data sparsity below 25°C, resulting in wider confidence intervals (Figure 4). Larger or targeted datasets may help refine estimates in this clinically critical regime.

Real-time deployment of GARIX for decision support will require careful treatment of latency and partial controllability in key variables. Hb and SaO_2_ are not truly exogenous; they reflect upstream physiological and clinical processes, such as transfusions or gas exchange, with inherent delays and feedbacks. Future extensions of the model should explicitly represent such dynamics to improve forecasting and responsiveness, and pave the way for closed-loop, physiology-informed perfusion control using GARIX as the core predictive engine.

This study did not examine associations between model-derived OG and clinical outcomes such as acute kidney injury (AKI) or neurologic events. Such analyses, while beyond the scope of the current work, are a critical next step in evaluating the prognostic utility of GARIX and its potential for informing goal-directed perfusion strategies.

For further discussion of the assumptions and limitations of GARIX – focused on the mathematical framework – see Part 1.

## Conclusion

In this second part of our series, we demonstrated how the GARIX model can generate clinically actionable insights from high-resolution intraoperative CPB data. By quantifying age- and weight-dependent oxygen demand, characterising nonlinear metabolic suppression across the temperature range, and revealing dynamic mismatches between oxygen supply and extraction, GARIX offers a physiologically grounded framework for personalising perfusion. These findings underscore the limitations of static assumptions – such as fixed flow targets and constant-Q_10_ models – and point toward more adaptive strategies that account for developmental stage, temperature regime, and temporal dynamics. As GARIX is further refined and validated, it holds promise not only as a descriptive tool but also as a foundation for real-time monitoring and intelligent perfusion control. Moreover, by demonstrating the clinical value of high-resolution intraoperative data for individualised goal-directed perfusion, this work highlights the importance of routine data capture and curation; wider adoption of such initiatives can create a virtuous cycle in which richer datasets enable more sophisticated and clinically useful GARIX models, further driving data-driven approaches and ultimately improving outcomes for paediatric patients undergoing CPB.

## Supplemental Material

Supplemental Material - Towards goal-directed perfusion - Part II: Temperature dependence of Q_10_, oxygen-demand dependence on age and weight, further clinical insights Supplemental Material for Towards goal-directed perfusion - Part II: Temperature dependence of Q_10_, oxygen-demand dependence on age and weight, further clinical insights by Mansour T. A. Sharabiani, Alireza S. Mahani, Richard W. Issitt, Yadav Srinivasan, Alex Bottle and Serban Stoica in Perfusion
