# Supplementary material for: Towards goal-directed perfusion - Part II: Temperature dependence of Q10, oxygen-demand dependence on age and weight, further clinical insights
Source: Perfusion. 2025 Oct 14;41(6):947–56. doi: 10.1177/02676591251385838 (PMC13490674; doi:10.1177/02676591251385838)
Supplement: Supplementary file 1 [file sj-pdf-1-prf-10.1177_02676591251385838.pdf]

Towards Goal-Directed Perfusion – Consolidated Supplementary Materials for Parts I & II

A: Nonstandard Abbreviations and Acronyms

- AKI: acute kidney injury
- ATG: autoregressive term group
- BSA: body surface area (m^2)
- bGARIX: Baseline GARIX where, in the equilibrium term group, a simple linear function of temperature replaces the temperature spline and the baseline demand's dependency on patient age and weight is removed.
- CI: cardiac index (blood flow indexed to BSA; $L/min/m^2$)
- CPB: cardiopulmonary bypass
- CV: cross-validation
- DO_2: oxygen delivery (mL/min)
- DO_2i: oxygen delivery, indexed to BSA ($mL/min/m^2$)
- ETG: equilibrium term group
- FTG: Future Term Group
- GARIX: A global autoregressive integrated (time-series) model with exogenous variables, plus a disequilibrium term group
- GDP: goal-directed perfusion
- Hb: haemoglobin content of blood (g/dL)
- Hct: haematocrit
- HLM: Heart-lung machine
- HTG: History Term Group
- i.i.d: independent and identically distributed
- KDIGO: Kidney Disease: Improving Global Outcomes

- LOESS: locally estimated scatterplot smoothing
- OER: oxygen extraction ratio
- OG: Oxygenation Gap, percentage difference between actual oxygen consumption (VO_2i) and target oxygen consumption (tVO_2i)
- OOS: out-of-sample
- PaO_2: partial pressure of oxygen in arterial blood (kPa)
- PvO_2: partial pressure of oxygen in mixed venous blood (kPa)
- PRAiS2: partial risk adjustment in surgery model
- PTG: Present Term Group
- PVI: permutation-based variable importance
- Q_{10} : multiplicative increase in the resting metabolic rate for every 10℃ increase in body temperature
- RBC: red blood cell
- RMR: resting metabolic rate
- SaO_2: oxygen saturation of arterial blood (%)
- SvO_2: oxygen saturation of mixed venous blood (%)
- Temp: Body temperature (°C)
- TG: term group, a collection of terms that additively contribute to minute-by-minute changes in the logit of OER in the GARIX and bGARIX models
- tVO_2i: target (indexed) oxygen consumption (aka indexed oxygen demand; $mL/min/m^2$)
- VI: variable importance
- VO_2: oxygen consumption (mL/min)
- VO_2i: oxygen consumption, indexed to BSA ($mL/min/m^2$)
- XTG: Exogenous Term Group

B: Study Design and Data Preparation

Study Population and Ethics

We conducted a retrospective analysis of intraoperative cardiopulmonary bypass (CPB) data collected at Great Ormond Street Hospital between 2019 and 2021. The study

population comprised all patients younger than 18 years of age—the maximum age treated at the institution—undergoing CPB during this period. Data were collated through the institution's electronic health record and intraoperative monitoring systems, then de-identified within a secure research platform under appropriate institutional approval (audit number 3045; REC reference: 17/LO/0008). Due to the retrospective and anonymised nature of the study, individual consent was not required.

Data Acquisition and Validation

Continuous minute-by-minute intraoperative data were recorded from the heart-lung machine and anaesthetic monitors, including cardiac index (CI, L/min/m^2), haemoglobin concentration (Hb, g/dL), haematocrit (Hct, %), arterial oxygen saturation (SaO_2, %), venous oxygen saturation (SvO_2, %), and body temperature (Temp, °C). Optical fluorescence and reflectance sensors (CDI550, Terumo) provided real-time blood gas measurements, calibrated every 20 minutes using cassette-based blood gas analysis (ABL90 Flex Plus, Radiometer). Although both Hb and Hct were available, we used only Hb when calculating the oxygen content of blood, as Hct is a derived quantity and highly correlated with haemoglobin. Additional clinical variables—including demographics, comorbidities, outcomes, and risk scores—were extracted via structured queries from the institutional electronic health record system.

Each surgical operation was segmented into one or more contiguous sessions of valid minute-level data. Data points were retained if they met the following criteria: $0 <$ haematocrit (Hct) $< 60\%$; $0\% < SvO_2 < SaO_2 < 100\%$; Temp > 0°C; CI > 0 L/min/m^2. Observations failing these filters or missing required values were excluded. Initial and terminal artefacts (e.g., CI = 0 at circuit initiation or termination) were removed. Only valid and contiguous time blocks were included for analysis.

Derived Variables

- Body Surface Area (BSA) was estimated using the Mosteller formula:

  $BSA = \sqrt{[(\text{height(cm)} \times \text{weight(kg)})/3600]}$.

- CI (Cardiac Index) is calculated by dividing pump flow (L/min) by the patient's BSA (m2). Thus CI has a unit of L/min/m2.
- Indexed Oxygen Delivery (DO_2 i) and Indexed Oxygen Consumption (VO_2 i) were computed using standard formulae that exclude dissolved oxygen:

$$DO_2\ i = 10 \times CI \times (1.34 \times Hb \times SaO_2\ /100) \quad (B.1)$$

$$VO_2\ i = 10 \times CI \times (1.34 \times Hb \times (SaO_2 - SvO_2)/100) \quad (B.2)$$

- Oxygen Extraction Ratio (OER) was calculated as:

$$OER = VO_2\ i\ /\ DO_2\ I = (SaO_2 - SvO_2)\ /\ SaO_2 \quad (B.3)$$

All calculations used haemoglobin-bound oxygen only, excluding the dissolved fraction, which accounted for ~1.6% of total arterial oxygen content and was considered negligible for modelling purposes.

Final Dataset and Exclusion Criteria

To ensure modelling reflected normative physiology, patients with postoperative acute kidney injury (AKI) were excluded from model training. AKI was defined using KDIGO criteria. The resulting training dataset comprised 293 CPB procedures across 286 patients, totalling 20,443 minutes of valid intraoperative data. This dataset was used to train both bGARIX and GARIX models.

Two specific analyses included all 963 patients: (1) benchmarking GARIX metabolic predictions against Schofield basal metabolic rate equations, and (2) empirical estimation of the joint age-weight distribution for response surface visualisations. This broader dataset included 926 patients with valid age and weight data and 60,260 minutes of data in total.

Model Variants and Training Subsets

Training datasets for different model variants were constructed using consistent validity criteria. When comparing GARIX(N) models with varying history lengths (N), the longest-history data subset (N=20) was used across all variants to ensure comparability of results (15,138 minutes of valid intraoperative data). For subgroup analyses by age, separate GARIX models were trained on patients stratified into four age groups: neonates (<1 month), infants (1–6 months), toddlers (6 months–3 years), and children (>3 years). Age was also treated continuously, and patient weight was log-transformed for modelling. For mathematical details of GARIX and bGARIX models, see Section D.

Software

All data processing and analysis were conducted using R 4.4. and its various open-source packages available on CRAN [7].

C: Patient Population and Clinical Outcomes

Of a total of 963 operations, there were 854 with known AKI status. Within those, 343 had no AKI, and 286/132/93 had AKI of 1/2/3, respectively. After filtering the intraoperative data (see Section B, Data Acquisition and Validation) we were left with 293 operations on 286 patients. Table C.1 shows data characteristics for the zero-AKI subset, which forms the training set for all GARIX and bGARIX models, with the exception of Figure 6B in Part 1. Most patients (66%) were 6 months or older, and gender distribution was balanced (45% female).

**Variable**	**Value**
Number of operations/patients	293/286
Total minutes of intraoperative readings<br>- Average # of minutes per operation	20,443<br>71
Median age at operation (months)	15.0 [4.3 – 57.7]

Range	0.1 – 217.4
Age group (at operation)	
Neonates (0-30 days)	43 (15)
Infants (1-6 months)	57 (19)
Toddlers (6 months -3 years)	96 (33)
Children (3-18 years)	97 (33)
Gender	
Male	156 (55)
Female	130 (45)
Median temperature (°C)	33.2 [31.3 – 34.7]
- range	15.9 – 36.8
OER	0.22 [0.16 – 0.27]
- range	0.01 – 0.66

Table C.1: Summary of data (AKI-free patients) used in training the GARIX and bGARIX models discussed in Parts 1 and 2 (except Figure 6B in Part 1). Numbers in brackets represent interquartile ranges for numeric variables, while numbers in parentheses represent percentages for discrete variables.

D: GARIX Mathematical Framework

Overview

We define two closely-related time-series models for predicting minute-by-minute changes in OER during CPB: a baseline model (bGARIX) and a full model (GARIX).

- bGARIX (baseline GARIX): Assumes a constant-Q_{10} relationship between temperature and metabolic demand and excludes any patient-specific covariates.

- GARIX**:** Extends GARIX by incorporating age, weight, their interaction, and a spline-based model of temperature dependence.

Both models share the same core structure, decomposing OER dynamics into three additive term groups:

1. Autoregressive Term Group (ATG)**:** Captures delayed self-correction in OER.
2. Exogenous Term Group (XTG)**:** Represents the effects of past and planned changes in CI, Hb, SaO_2, and Temp.
3. Equilibrium Term Group (ETG)**:** Encodes the corrective force that aligns oxygen consumption with metabolic demand (tVO_2i).

The following assumptions underpin GARIX:

- Linearity of effects in all terms (except spline-based temperature dependence in GARIX).
- Gaussian, homoscedastic noise.
- Exogenous control of CI, Hb, SaO_2, and Temp.
- bGARIX: constant-Q_{10}, no patient covariates.
- GARIX: spline-based Q_{10}, patient-specific demand scaling.
- Dissolved oxygen is excluded from DO_2i and VO_2i calculations.

Figure D.1 is a “model card” for GARIX, summarising its aims, key components and assumptions.

What does GARIX stand for?

- GARIX: Global AutoRegressive Integrated time-series model with eXogenous variables and an 'equilibrium' force
- The equilibrium force represents the gap between oxygen consumption and oxygen demand, with logarithm of oxygen demand being a function of patient's age, weight and body temperature.

What is the aim of GARIX? Predicting near-term changes in OER during CPB as a function of *system history*, *present state*, and *intended actions* (perspective #1):

- *System history ('past')*: Past changes in CI, Hb, SaO_2, temperature and OER (HTG)
- *Present state ('present')*: Current values of CI, Hb, SaO_2, temperature and OER (ETG)
- *Intended actions ('future')*: Planned changes in CI, Hb, SaO_2 and temperature (FTG)

Alternative interpretation - GARIX model describes OER changes as a function of *autoregressive*, *equilibrium*, and *exogenous* forces (perspective #2):

- *Equilibrium* force seeks to change oxygen consumption (VO_2i) - via changing OER - to ensure it matches oxygen demand (tVO_2i), as dictated by temperature. (ETG)
- *Autoregressive* force serves to reverse random fluctuations in OER, thus creating further stability when system is near equilibrium. (ATG)
- *Exogenous* force reflects short-term impact of changes in CI, Hb, SaO_2 and temperature on OER to keep VO_2i aligned with tVO_2i. (XTG)

The GARIX model maps each of the three components in Perspectives #1 and #2 to a collection of regression terms, referred to as term groups (TGs) in the text.

Connection between perspectives:

- Present state of system is summarised by the equilibrium force (ETG).
- The sum of history and future term groups is equal to the sum of autoregressive and exogenous term groups, i.e., HTG + FTG = ATG + XTG.

Model scope and idealisations:

- Model is global, i.e., parameters are shared across all patients (homogeneity assumption).
- Focus is on haemoglobin-bound oxygen (excluding dissolved oxygen).
- CI, Hb and SaO_2 are considered exogenous, i.e., fully controllable by the perfusionist.
- System noise is homoscedastic, i.e., random fluctuations in (logit of) OER always have the same overall strength and across all patients.

Figure D.1: Conceptual summary of the GARIX model for predicting OER changes during paediatric CPB, proposed in this paper.

Autoregressive and Exogenous Term Groups

Let $\Delta logit(OER_t)$ denote the change in logit-transformed OER from time t to t+1. Recall the main equation in GARIX:

$$\Delta logit(OER_t) = ATG_t + XTG_t + ETG_t + \epsilon_t, \tag{D.1}$$

where

- $\epsilon_t \sim N(0, \sigma^2)$ is the noise term, which is an independent draw at time t from a Gaussian distribution with variance σ^2,
- The logit function is defined as logit(x) = log(x / (1 - x)),
- $\Delta logit(OER_t) = logit(OER_{t+1}) - logit(OER_t)$.

ATG is given by:

$$ATG_t = \sum_{n=1}^{N} \alpha_n \Delta logit(OER_{t-n}), \tag{D.2}$$

where N is the maximum lag or history length (a tuning parameter of GARIX), and α_n's are autoregressive coefficients that will be estimated from data.

XTG is given by:

$$XTG_t = \sum_{n=0}^{N} \sum_X \beta_n^X \Delta X_{t-n}, \tag{D.3}$$

with $X \in \{\log(CI), \log(Hb), \log(SaO_2), Temp\}$. As with the ATG, we have $\Delta X_t = X_{t+1} - X_t$. β_n^X's are also model coefficients estimated from data.

This term captures the effects of past (n > 0) and intended (n = 0) minute-level changes in exogenous/controllable perfusion parameters on OER.

Note the difference between ATG and XTG in terms of the range of n's used in the sums. While the XTG includes n = 0, ATG does not. This is because OER is an endogenous variable, whose future value – OER_{t+1} – we are seeking to predict at time t. In other words, while the values of exogenous variables at t + 1 are assumed to be known – or

more precisely, controllable by perfusionist – at time t, the t+1 value of OER is unknown at time t and its prediction is the goal of the GARIX model.

History and Future Term Groups

Alternatively, the sum of ATG and XTG can be decomposed into history and future term groups – HTG and FTG, respectively.

HTG represent the contribution of past changes to OER from all system variables including controllable ones (CI, Hb, SaO2 and Temp) as well as OER itself:

$$HTG_t = \sum_{n=1}^{N} \sum_X \beta_n^X \, \Delta X_{t-n}, \tag{D.4}$$

with $X \in \{\log(CI), \log(Hb), \log(SaO_2), Temp, logit(OER)\}$. Note that, in contrast to XTG, here X includes logit(OER), but also that the index n starts at 1 here.

FTG represents the effect of intended actions – or changes from t to t+1 - in the controllable variables only:

$$FTG = \sum_X \beta_0^X \Delta X_t, \tag{D.5}$$

Equilibrium Analysis

Recall the definition of ETG:

$$ETG_t = \kappa \times [\log(VO_2 i_t) - \log(tVO_2 i_t)], \tag{D.6}$$

where $VO_2 \; i_t$ is the observed indexed oxygen consumption (Equation B.2), and $tVO_2 \; i_t$ is the latent metabolic demand.

To see why we are justified to interpret tVO_2 i as the latent oxygen demand, assume the system is in perfect equilibrium, i.e., all system measurements (CI, Hb, SaO_2, Temp and OER) remain unchanged over time. Consequently, the following conditions apply for all t:

1. ΔOER = 0,
2. ATG = 0 (no autoregressive drift),
3. XTG = 0 (no net effect from recent or ongoing changes in exogenous variables).

Combining the above three conditions with Equation D.1, and noting that noise is assumed to be zero-mean, we conclude that ETG must be zero. From Equation D.4, ETG = 0 implies that:

$$\log(VO_2\ i) = \log(tVO_2\ i) \Longrightarrow VO_2\ i = tVO_2\ i. \quad (D.7)$$

If we further assume that the body's goal is to match oxygen consumption to its demand, we conclude that during 'equilibrium', consumption and demand must be equal. But from Equation D.5, consumption (VO_2 i) equals tVO_2 i. Therefore, tVO_2 i can be interpreted as oxygen demand.

In a trained GARIX model, steady-state analysis can be used to calculate the equilibrium OER, given patient age and weight, and the target CI, Hb, SaO_2 and Temp. More specifically:

1. Given patient age, weight and target Temp, we calculate tVO_2 i (see below).
2. Assuming a system in equilibrium, we set VO_2 i equal to the calculated $tVO_2\ i_t$.
3. Since $VO_2\ i = 10 \times CI \times (1.34 \times Hb \times SaO_2\ / 100) \times OER$, we plug in the target values for CI, Hb and SaO_2, and solve for OER.

An identical approach can be used to solve for any of CI, Hb, and SaO_2 if other variables including OER are known.

To solve for Temp in equilibrium analysis, we follow these steps:

1. Given CI, Hb, SaO_2 and OER, calculate VO_2 i.
2. Assuming equilibrium, set tVO_2 i equal to VO_2 i.
3. Insert patient's age and weight into the expression for tVO_2 i (see below), and solve for Temp.

Modelling $tVO_2 i$

The distinction between bGARIX and GARIX lies in the mathematical specification of the $tVO_2 i$ term.

In bGARIX, we assume the following functional form:

$$\log(tVO_2 i) = \gamma_0 + \gamma_1 \times \text{Temp}. \quad \text{(D.8)}$$

This is the classical van't Hoff form: the logarithm of oxygen demand increases linearly with temperature, corresponding to a constant Q_{10} across all temperatures and patients:

$$Q_{10} = \exp\left(10 \times \frac{\delta \log(tVO_2 i)}{\delta T}\right) = \exp(10\,\gamma_1). \quad \text{(D.9)}$$

On the other hand, GARIX implies the following function for oxygen demand:

$$\log(tVO_2 i) = \gamma_0 + \gamma_1 \log(\text{age}) + \gamma_2 \log(\text{weight}) + \gamma_3 \log(\text{age}) \times \log(\text{weight}) + s(\text{Temp}) \quad \text{(D.10)}$$

We can see from Equation D.8 that GARIX allows the intercept of the oxygen demand (as a function of temperature) to vary with patient-specific factors (age and weight). It also replaces the linear temperature term with a smooth spline s(Temp), enabling Q_{10} to become a function of temperature. (Note that Q_{10} will not be dependent on patient's age or weight.)

We calculate local and interval Q_{10}'s for GARIX by numerical approximation:

$$Q_{10}^{local}\left(\frac{T_i+T_{i+1}}{2}\right) \approx \exp(10 \times \frac{\log\left(tVO_2 i(T_{i+1})\right)-\log\left(tVO_2 i(T_i)\right)}{T_{i+1}-T_i}), \quad \text{(D.11)}$$

The above approximately corresponds to the slope of the tangent to the $\log(tVO_2 i)$-Temp curve at $(T_i + T_{i+1})/2$. For interval Q_{10}:

$$Q_{10}^{interval}(T) = \exp(10 \times \frac{\log(tVO_2 i(37)) - \log(tVO_2 i(T))}{37 - T}). \quad \text{(D.12)}$$

The above corresponds to the slope of the line connecting the (Temp, log(tVO_2 i)) points between T and 37. Please note that, unlike bGARIX where Q_{10} is a constant, in GARIX both local and interval Q_{10}'s are functions (of temperature).

System Simulations

As a time-series model, GARIX can be used – after training – to 'forecast' OER. In this context, we refer to it as 'system simulation'. This proceeds in one-minute time steps, with a single-step simulation at time t characterised below:

- Inputs:
    - History and Present: These are current and past values of controllable variables (CI, Hb, SaO_2 and Temp) and OER. For example, for CI we need $CI_{t-5}, CI_{t-4}, CI_{t-3}, CI_{t-2}, CI_{t-1}$ and CI_t, and similarly for other variables.
    - Actions: These are intended values of controllable parameters at next time step, i.e., t+1: $CI_{t+1}, Hb_{t+1}, SaO_{2_{t+1}}, Temp_{t+1}$. Note that OER at t+1 is unknown and hence is not an input to the model but rather its output.
- Output:
    - OER_{t+1}
- Simulation steps (trained GARIX acting on inputs):
    1. Calculate ATG_t, XTG_t, ETG_t, or alternatively HTG_t, FTG_t, ETG_t.
    2. Generate ϵ_t, a random draw from a zero-mean Gaussian with variance σ^2.
    3. Use Equation D.1 to calculate $\Delta logit(OER)_t$.
    4. Add $\Delta logit(OER)_t$ to $logit(OER)_t$ to obtain $logit(OER)_{t+1}$.
    5. To obtain OER_{t+1}, reverse the logit transformation: $OER_{t+1} = 1/(1 + exp(-logit(OER)_{t+1}))$.

The above can be repeated as many times as needed to simulate the system over longer time intervals. Note that actions at all future times must be supplied as input.

There is a special case where controllable variables (CI, Hb, SaO_2 and temp) are left unchanged, and system is allowed to evolve for many time steps towards equilibrium. At equilibrium, Equation D.7 must be satisfied which, combined with Equation B.3, means we must have:

tVO_2 i = DO_2 i × OER

(D.13)

For the above to be feasible, and given that OER = (SaO_2 − SvO_2) / SaO_2 <= 1.0, we must have

tVO_2 i <= DO_2 i

(D.14)

where DO_2 i can be calculated based on CI, Hb, SaO_2 using Equation B.1. If the above condition is note satisfied, then the system will not reach true equilibrium but OER will approach 1.0 asymptotically (logit of OER will grow unboundedly positive).
There are physiological reasons to think that OER above 0.5-0.6 is toxic and body's behaviour may drastically change when OER crosses these critical values. Given this, and the fact that our training data for GARIX contained OER values below this critical region, we should take care not to 'extrapolate' from our data by conducting system simulations in high-OER regions.

Where the objective is to study "average" system behaviour, it is more efficient to switch off the noise and run the simulations in "deterministic" mode, rather than running 'stochastic' simulations many times and taking their average. The only difference between the two simulation modes is whether ϵ_t, which is a random draw from a

Gaussian with variance based on GARIX model's residuals, is generated and added to the deterministic component of OER or not (step #2 of simulation steps listed earlier).

E: Model Diagnostics

The bGARIX model was fit using standard ordinary least squares (OLS) regression, while the full GARIX model – due to its inclusion of spline terms – was estimated via backfitting using the gam function from the R package gam [6].

We performed autocorrelation and heteroscedasticity checks on the model residuals (Figure E.1). Residuals were computed as the difference between observed and predicted Δlogit(OER) on the training data.

- **Autocorrelation**: Combined autocorrelation functions (ACF) were computed across all residual segments. The "combined" ACF pools lag correlations from all CPB runs to assess whether residuals exhibit systematic time dependencies. The plot (panel A) closely resembles the random noise version (panel B), suggesting that differencing of OER and exogenous terms has been sufficient.
- **Heteroscedasticity**: To assess variance stability, we examined the ACF of the absolute residuals. Systematic autocorrelation in this series (panel C) compared to rando noise (panel D) suggests that the magnitude of residual magnitude varies over time, a hallmark of heteroscedasticity.

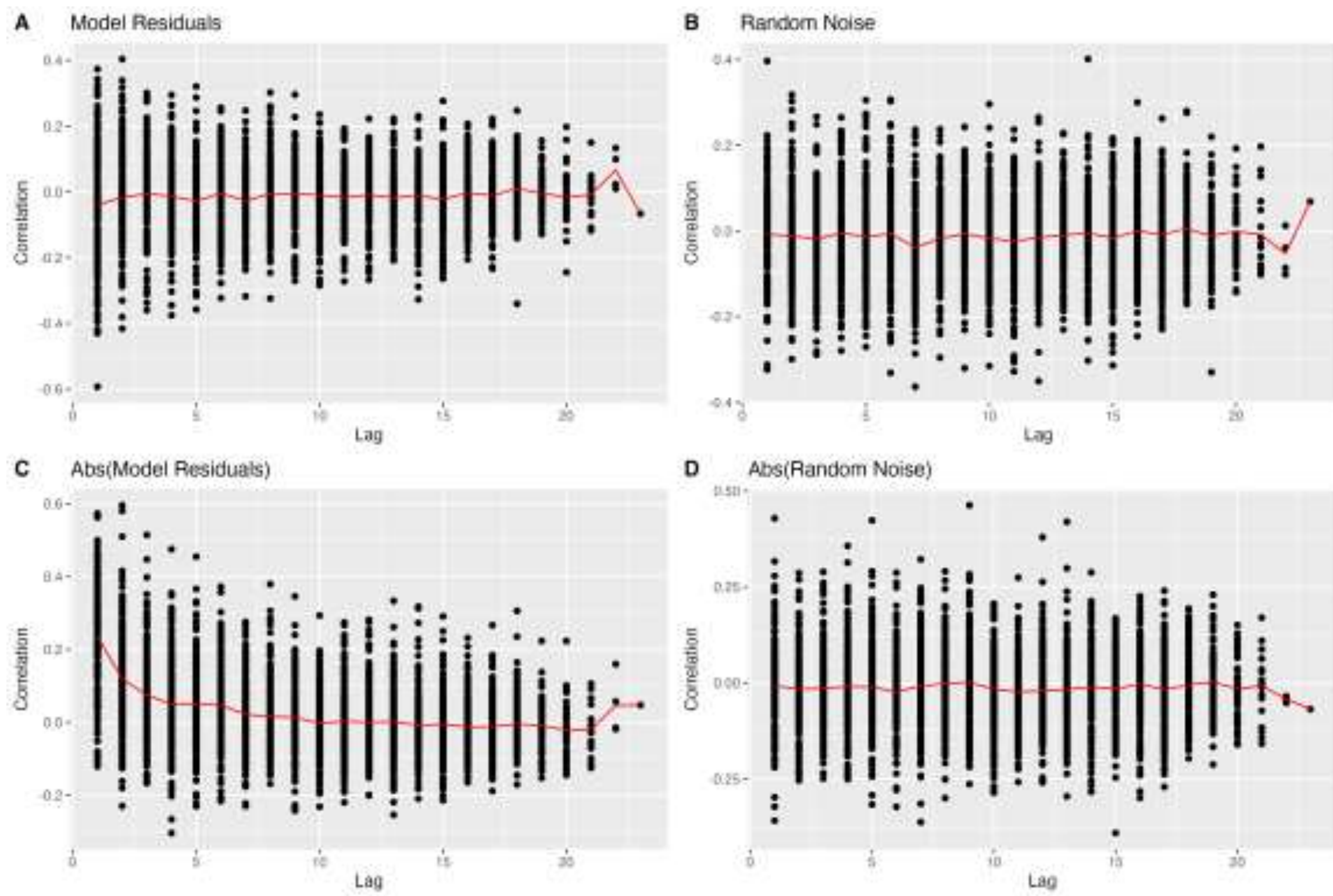

Figure E.1: Autocorrelation analysis of GARIX model residual noise. A: Combined autocorrelation plot of GARIX(5) residuals. B: Combined autocorrelation plot of random Gaussian noise for an identical time-series collection. C: Combined autocorrelation plot of the absolute values of model residuals. D: Combined autocorrelation plot of the absolute values of Gaussian noise.

F: Additional Results

Age/Weight Interaction

The GARIX model incorporates an interaction term between log(age) and log(weight) to allow the relationship between weight and oxygen demand to vary with developmental stage. This interaction term was found to be statistically significant, indicating that weight does not exert a uniform effect on indexed oxygen demand (tVO_2 i) across ages. To visualise this relationship (Figure F.1), we examined the association between patient weight and predicted tVO_2 i at a fixed temperature (35 °C), across four representative

ages spanning the paediatric spectrum: 5-day-old neonate, 3-month-old infant, 1-year-old toddler, and 8-year-old child.

Weight values were generated over a ±2 standard deviation range around the smoothed log-weight distribution at each age. These distributions were estimated using LOESS fits to observed data, and GARIX predictions were generated for each point on the age-specific weight grids. The resulting demand curves illustrate how the marginal effect of weight on oxygen demand varies with age. For example, at early ages, higher weight is associated with higher indexed demand, whereas in older children the relationship becomes flat or even negative – indicating that heavier children in this group may exhibit lower tVO_2 i, possibly due to body composition changes.

These findings support the inclusion of age-weight interaction in demand modelling and emphasise the need for jointly personalised perfusion strategies based on both size and developmental stage.

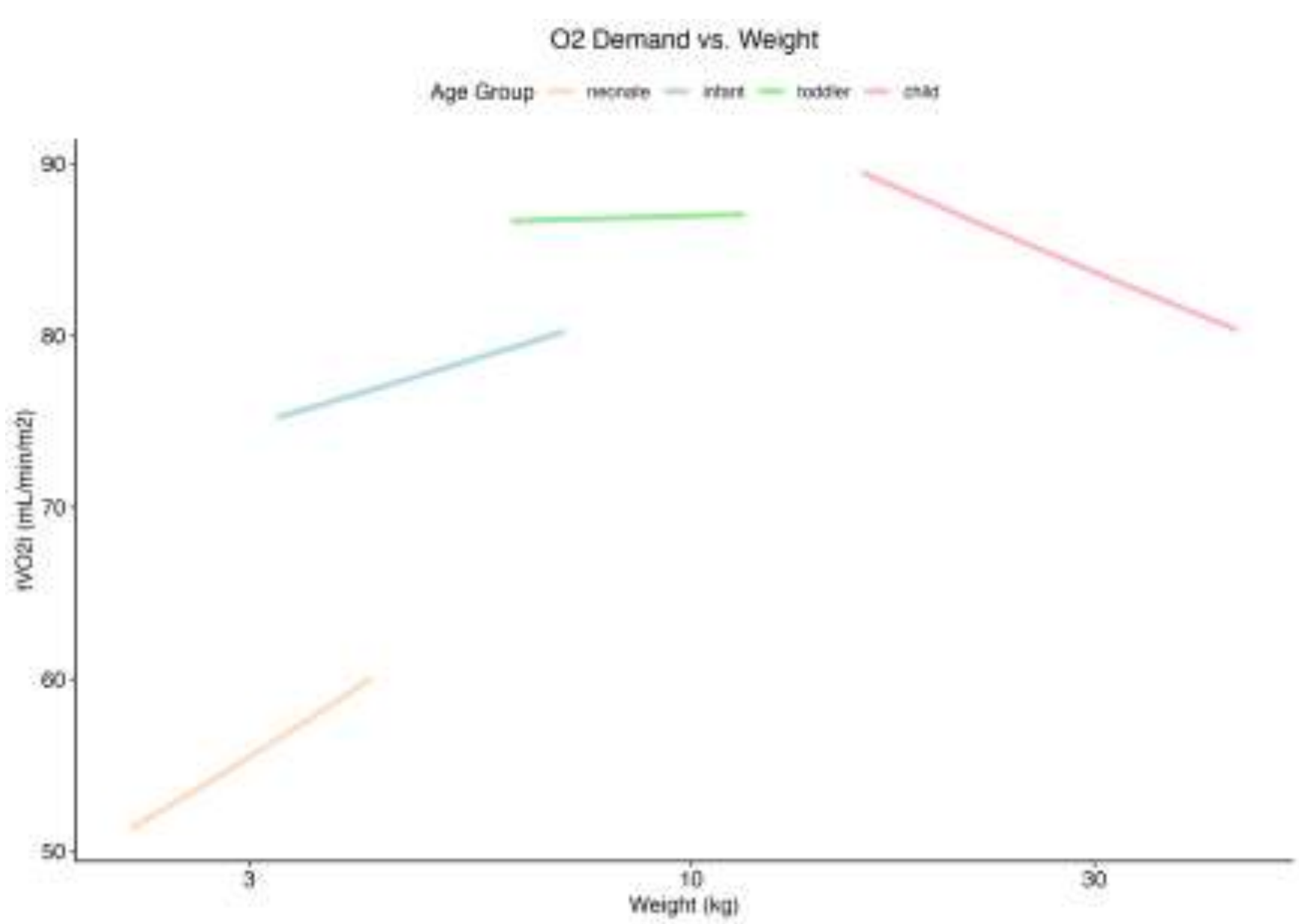

Figure F.1: Indexed Oxygen Demand (tVO_2 i) vs. Patient Weight at Representative Ages. Predicted tVO_2 i at 35 °C across a range of body weights, evaluated separately at four representative ages: 5-day-old neonate, 3-month-old infant, 1-year-old toddler, and 8-year-old child. Weight values were generated over a ±2 standard deviation range around

the smoothed log-weight distribution for each age, using LOESS fits to the training cohort. Each line shows the GARIX-predicted relationship between weight and tVO_2 i at the given age. Colours indicate age group as per the manuscript's age stratification. Note the changing slope across ages, indicating an interaction between age and weight in determining oxygen demand.

Coefficients by Age Group

Figure F.2 shows lagged-difference coefficients for all variables in GARIX. The haemoglobin plot (panel A) was discussed in Part 2. We also see a pattern for SaO_2 , where the lag = 0 coefficients are larger in infants and neonates compared to the older patients. As mentioned in Part 1 (Table 1), the positive coefficient at lag = 0 for SaO_2 is partly due to it being part of OER definition: OER = (SaO_2 - SvO_2) / SaO_2 . The following negative coefficients at larger lags reflect adaptive response to lower consumption. Stronger early response in neonates and infants may be caused by a weaker adaptive response that has not masked the instantaneous positive response.

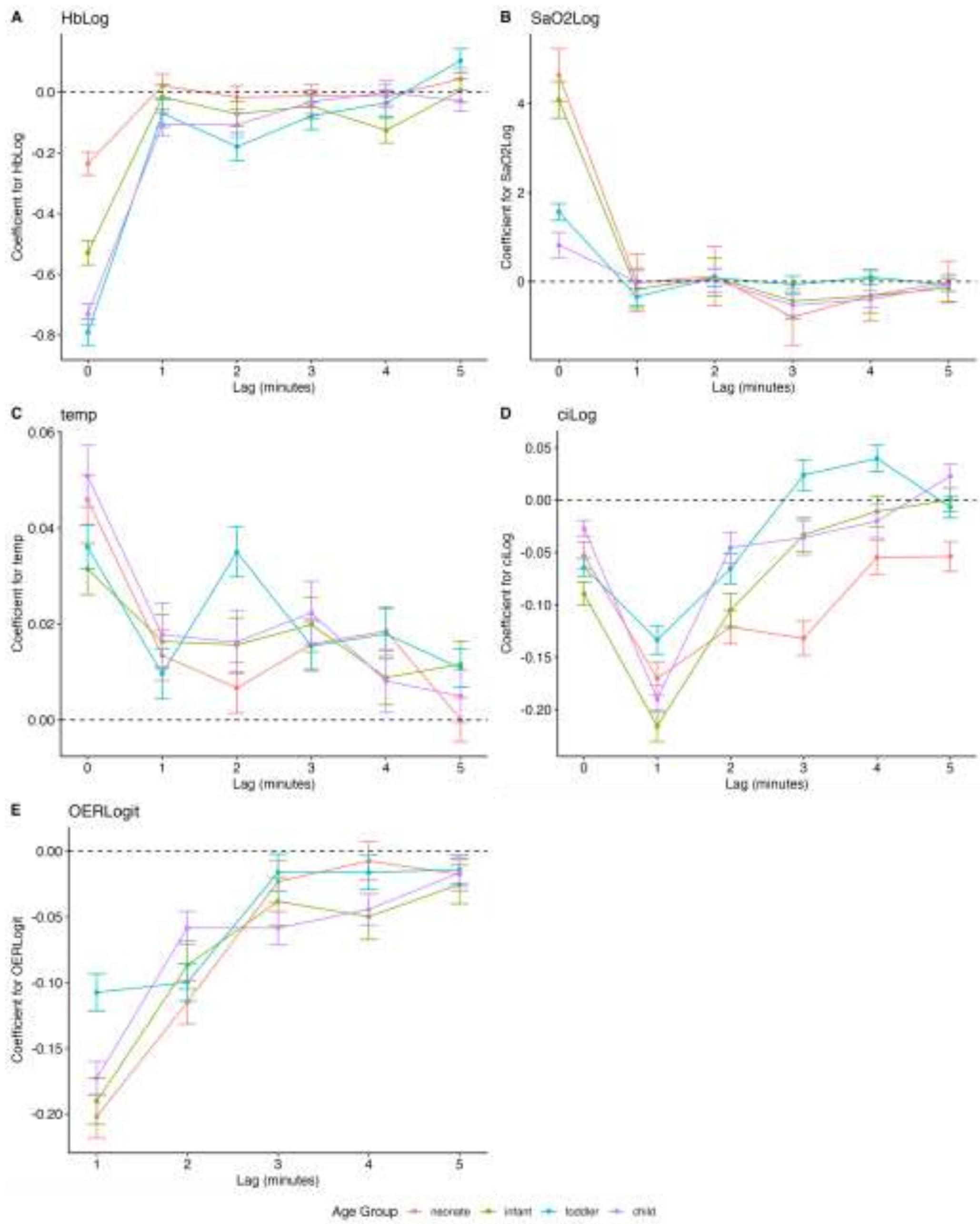

Figure F.2: Coefficients of lagged-difference terms – as a function of lag – in GARIX for all system variables. For each age group, GARIX was trained independently on the subset of data with patients restricted to that age group only.

G: Oxygen Demand in Kirklin's Textbook

The cardiac surgery textbook of Kirklin et al [1] recommends a hyperbolic function to describe the dependence of indexed oxygen consumption on cardiac index (and oxygen

delivery), and a van't Hoff function for the dependence of the two parameters of the hyperbolic function on temperature, which implies a constant Q_{10}. Below is a reproduction of Appendix Equation 2A-3 in [1]:

$$\frac{1}{tVO_2i} = 0.168 \times 10^{-0.0387 \times Temp} + 0.0378 \times \left(\frac{1}{CI}\right) \times 10^{-0.0253 \times Temp} \tag{F.1}$$

This equation is based on two sets of experiments. First is a series of animal experiments conducted by Cheng et al. [2], Paneth et al [3], and Starr [4], all conducted at 37°C temperature. Second is the human experiments conducted by Fox et al. [5], conducted at 20°C temperature.

Oxygen demand can be interpreted as tVO_2i when CI grows very large. This leads to a van't Hoff relationship between tVO_2i and $Temp$:

$$tVO_2i = \frac{10^{0.0387 \times Temp}}{0.168} \tag{F.2}$$

which implies a Q_{10} of 2.4.

Since infinite CI is impractical, Kirklin et al provide 'clinical' values for CI and the associated tVO_2i levels (Figure 2-11 of the textbook) for several temperatures. We extracted them through scanning and image analysis of the figure: 25, 45, 65, 120 (all in mL/min/m2) for temperatures of 20, 25, 30, 37 respectively.

Kirklin also presents a second van't Hoff model (Figure 2-1 and Appendix Equation 2A-1 in [1]):

$$tVO_2i = 10^{-0.69 + 0.043 \times Temp} \tag{F.3}$$

This implies a Q_{10} of 2.7.

In Figure 4 of Part 2, we refer to Equations F.2 and F.3 as ‘Kirklin-1’ and ‘Kirklin-2’, respectively.

H: Fox et al.’s Human Experiments

Below are further details on the Fox et al.’s human experiments [5], published in 1982:

A total of 17 adults (15 males, 2 females) were included whose age was 57+/-10 years (mean +/- std). Mean body temperature during recordings was 21.3C. Initial CI was 2.2 L/min/m2. Mean venous Hb was 7.1. SaO_2 was not given, but we assumed a value of 99%, consistent with their assertion that 100% oxygen (no CO2) was used for ventilator oxygenation. After an initial stabilisation period, a randomly-shuffled sequence of five nominal CI’s (0.25, 0.5, 1.0, 1.5 and 2.0 L/min/m2) were used with each patient, holding each level for 10 minutes. After device recalibration, actual flows were estimated to be 0.25 +/- 0.084, 0.54 +/- 0.101, 1.02 +/- 0.107, 1.56 +/- 0.129 and 2.08 +/- 0.180. Due to surgical constraints, the average number of 10-min episodes used with each patient was 4 (rather than 5).

We used the above mean and std values to draw CI’s for our simulations. Note the contrast between the age range of subjects in the above experiments vs. the paediatric population comprising our dataset.

I. Approximate Equivalence of van’t Hoff and Arrhenius Specifications

The Arrhenius and van’t Hoff formulations are the best-known parametric forms used to describe the relationship between temperature and oxygen consumption. It can be demonstrated that, if temperature changes are small compared to their absolute values, when expressed in Kelvins, the two specifications are approximately equivalent. In this manuscript, the van’t Hoff specification has been adopted, which is consistent with the widely recognized concept of Q_{10}, the multiplicative increase in resting metabolic rate for every 10°C increase in body temperature.

The Arrhenius equation, after mapping the concept of chemical reaction rate to metabolism and hence oxygen consumption, would require the logarithm of oxygen consumption to be a linear function of the inverse of temperature, expressed in Kelvins (T_K):

$$log(VO_2i) = a + b/T_K \quad \text{(E.1)}$$

Let's express T_K in terms of its deviations (ΔT) from a baseline value, T_0:

$$T_K = T_0 + \Delta T. \quad \text{(E.2)}$$

Equation D.1 can be rewritten as:

$$log(VO_2i) = a + b/(T_0 + \Delta T) \quad \text{(E.3)}$$

If $\Delta T << T_0$, i.e., if changes in temperature are small compared to its baseline value, in Kelvins, then a first-order, Taylor-series expansion is used to rewrite Equation (D.3):

$$log(VO_2i) = a + \frac{b}{T_0\left(1+\frac{\Delta T}{T_0}\right)} \sim a + (b/T_0)(1 - \Delta T/T_0) = (a + b/T_0) + (-b/T_0^2)\, \Delta T$$

(E.4)

Note that the last expression on the right is a linear function of temperature change, ΔT. Recalling – from Equation D.2 – that ΔT itself is a linear function of T_K, this is simply the van't Hoff specification, i.e., logarithm of VO2i being a linear function of temperature (expressed in Kelvins or any other units).

References

1. Kirklin JK, Blackstone EH. Kirklin/Barratt-Boyes Cardiac Surgery: Expert Consult-Online and Print (2-Volume Set). Elsevier Health Sciences; 2012 Oct 26.

2. Cheng HC, Kusunoki T, Bosher Jr LH, McElvein RB, Blake DA. A study of oxygen consumption during extracorporeal circulation. ASAIO Journal. 1959 Apr 1;5(1):273-8.
3. Paneth M, Sellers R, Gott VL, Weirich WL, Allen P, Read RC, Lillehei CW. Physiologic studies upon prolonged cardiopulmonary by-pass with the pump-oxygenator with particular reference to (1) acid-base balance,(2) siphon caval drainage. Journal of Thoracic Surgery. 1957 Nov 1;34(5):570-9.
4. Starr A. Oxygen consumption during cardiopulmonary bypass. The Journal of Thoracic and Cardiovascular Surgery. 1959 Jul 1;38(1):46-56.
5. Fox LS, Blackstone EH, Kirklin JW, Stewart RW, Samuelson PN. Relationship of whole body oxygen consumption to perfusion flow rate during hypothermic cardiopulmonary bypass. The Journal of Thoracic and Cardiovascular Surgery. 1982 Feb 1;83(2):239-48.
6. Hastie T (2024). gam: Generalized Additive Models. R package version 1.22-5, <https://CRAN.R-project.org/package=gam>.
7. R Core Team. *R: A Language and Environment for Statistical Computing*. Version 4.x, R Foundation for Statistical Computing, 2024, https://www.R-project.org/.
